# Supplementary material for: Selective Assembly of TRPC Channels in the Rat Retina during Photoreceptor Degeneration
Source: Int J Mol Sci. 2024 Jun 30;25(13):7251. doi: 10.3390/ijms25137251 (PMC11242081; doi:10.3390/ijms25137251)
Supplement: Supplementary file 1 [file ijms-25-07251-s001.zip › Fig. S4_ Supplemental Figure S4.pdf]

Fig. S4

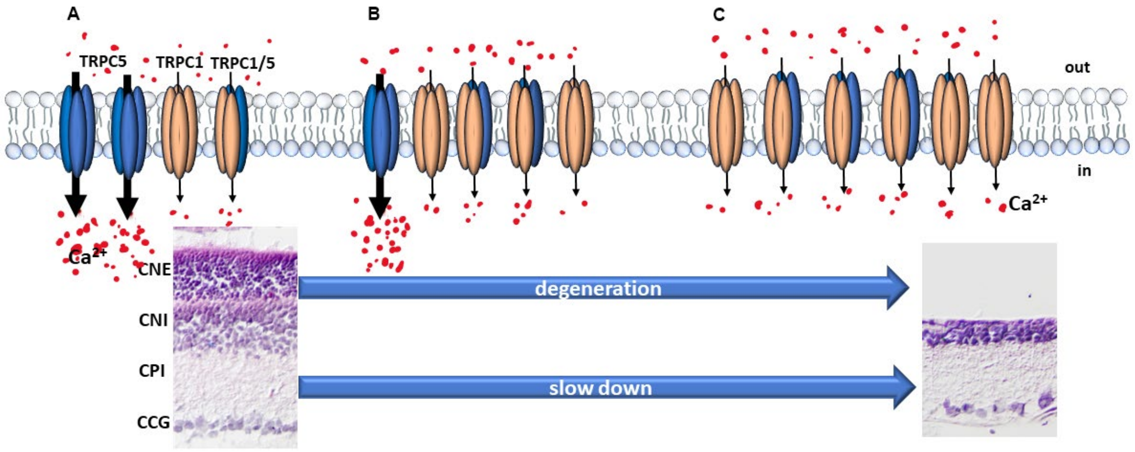

Figure S4. A model of the effects of the TRPC1/5 heteromer increase in retinas with progressive degeneration of photoreceptors. The results of studies on neurodegenerative diseases such as glaucoma, Parkinson's disease, or Huntington's disease, both in patients and in experimental models, provide support for the approach presented here after analyzing our results in the retinas of P23H rats with retinitis pigmentosa (RP). The TRPC1 channels are involved in the flow of calcium in the retina through different mechanisms, depending on the cell type. The loss of photoreceptors due to RP results in an increase in TRPC1 channel expression and increase of TRPC1 and TRPC5 interaction to form TRPC1/5 heteromers (A→C). The maintenance of elevated intracellular calcium concentrations without toxicity is essential for the continued activity of the inner retina, which is responsible for the circadian cycles and the maintenance of visual cortex activity, among other functions. It is therefore plausible to suggest that TRPC1, when interacting with TRPC5, influences the flow of calcium inside the cells of the inner retina. This may result in calcium levels that are less toxic than those that could be found if TRPC5 were to remain functioning as homomers.
