# Supplementary material for: Selective Assembly of TRPC Channels in the Rat Retina during Photoreceptor Degeneration
Source: Int J Mol Sci. 2024 Jun 30;25(13):7251. doi: 10.3390/ijms25137251 (PMC11242081; doi:10.3390/ijms25137251)
Supplement: Supplementary file 1 [file ijms-25-07251-s001.zip › Figs. S1-S3_Supplemetal Figures S1-S3.pdf]

# Fig. S1

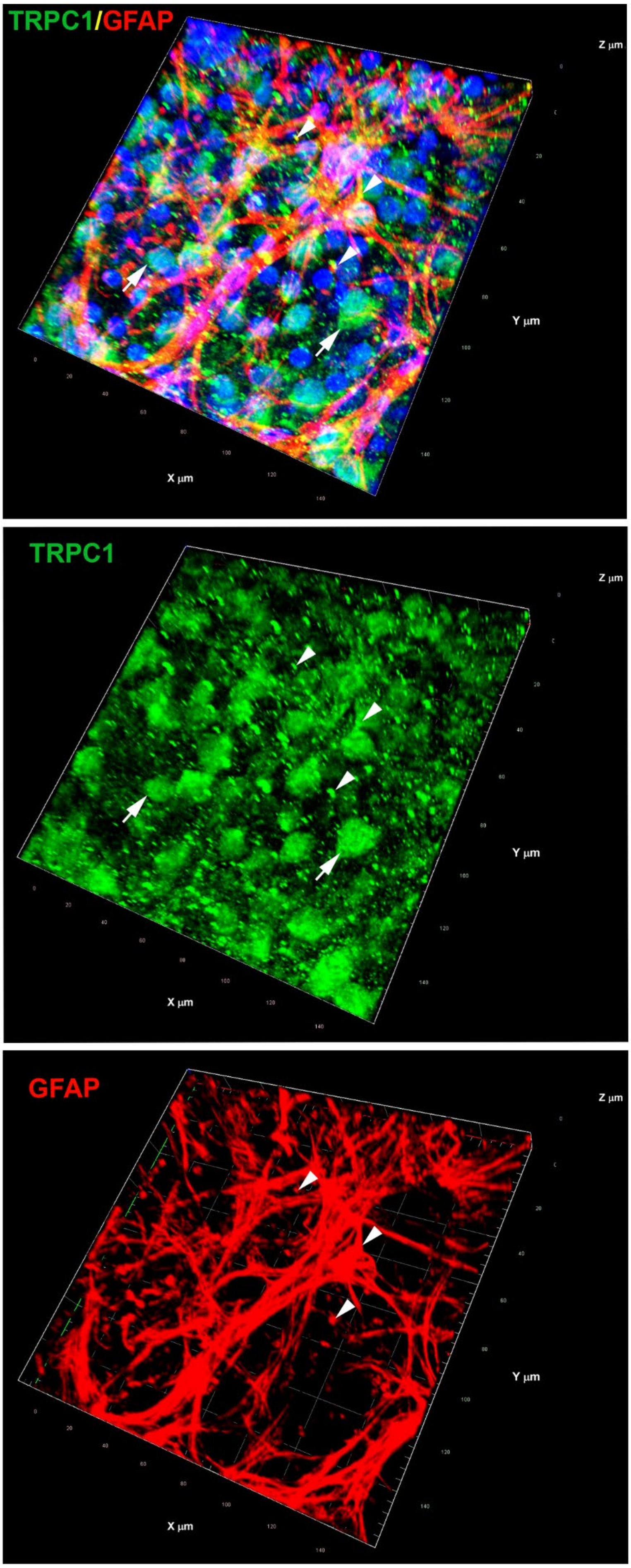


**Figure S1**. **Immunolocalization of TRPC1 (green) and GFAP (red) in a P23H-P240 rat retina.** 3D composition reconstructed from 10 scanned images taken from a whole mounted retina. Overlap in yellow. Overlap in glial cells (arrowheads) and localization of TRPC1 in ganglion cells (arrows).

# Fig. S2

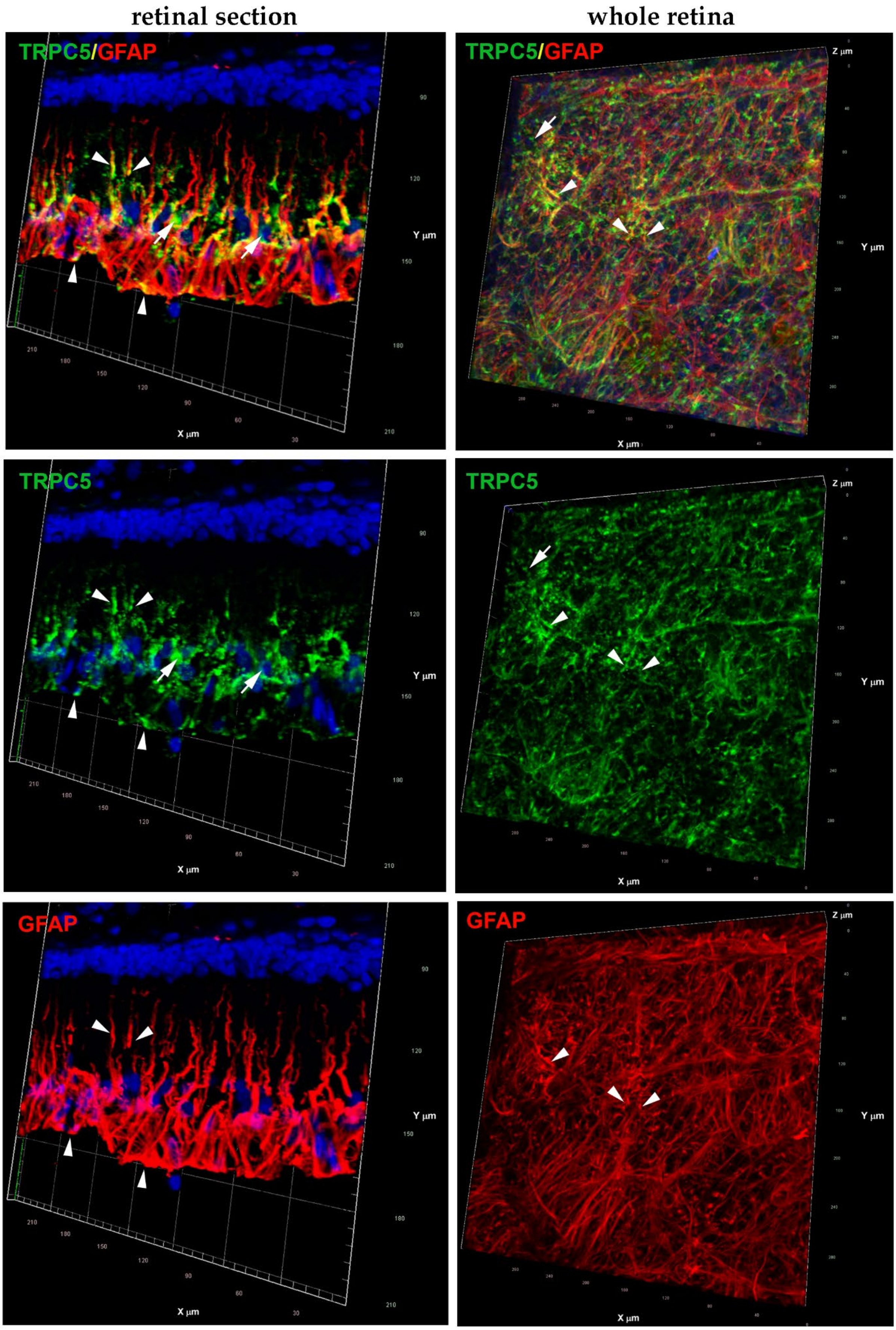


**Figure S2**. **Immunolocalization of TRPC5 (green) and GFAP (red) in a P23H-P240 rat retina.** 3D composition reconstructed from 10 scanned images taken from a retinal section (column left) and from a whole mounted retina (column right). Overlap in yellow. Overlap in glial cells (arrowheads) and localization of TRPC5 in ganglion cells (arrows).

# Fig. S3

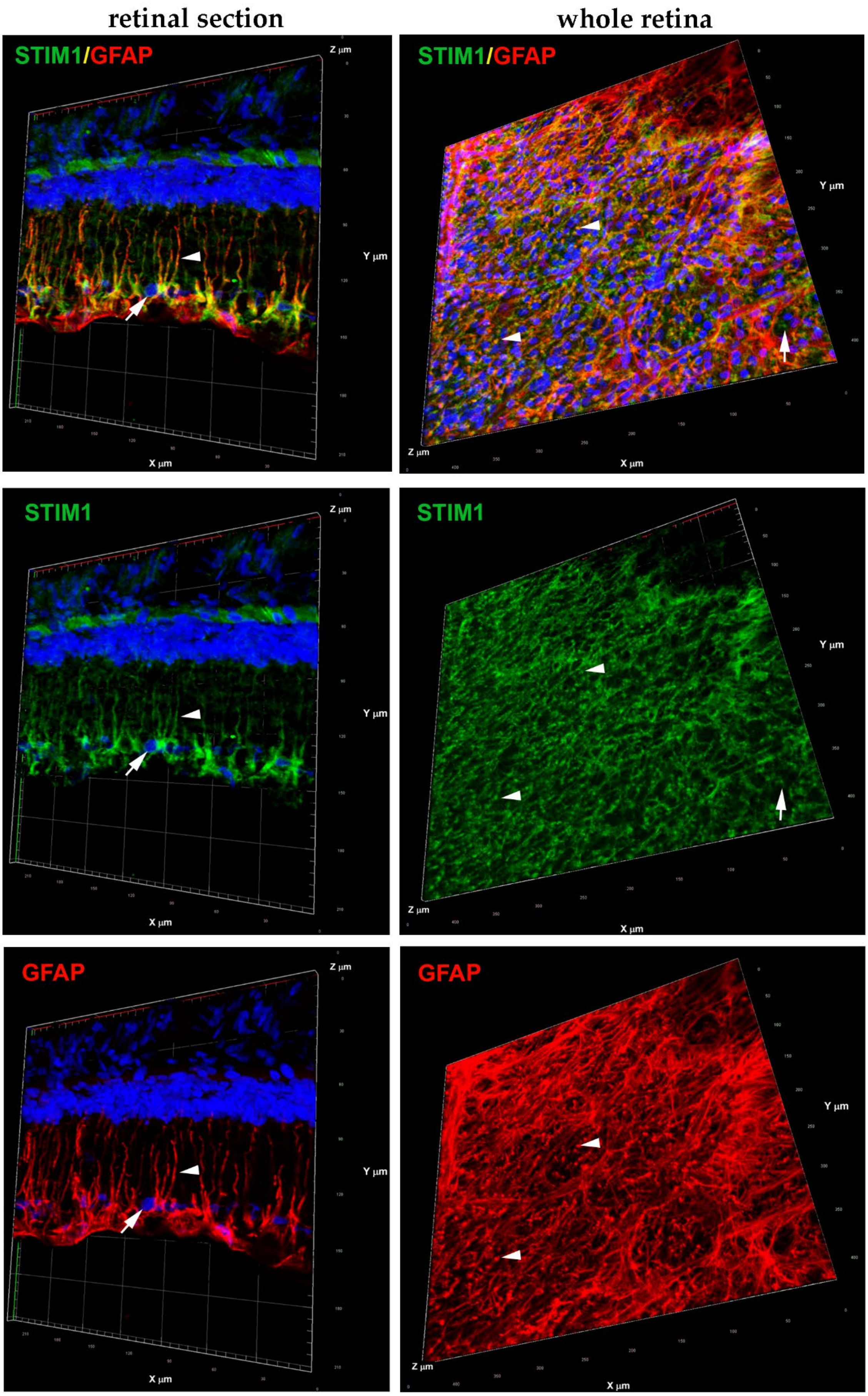


**Figure S2. Immunolocalization of STIM1 (green) and GFAP (red) in a P23H-P240 rat retina.** 3D composition reconstructed from 10 scanned images taken from a retinal section (column left) and from a whole mounted retina (column right). Overlap in yellow. Overlap in glial cells (arrowheads) and localization of TRPC5 in ganglion cells (arrows).
